# Defining the ATA-2025 ‘consider RAIT’ zone in older patients with N1b PTC

**DOI:** 10.1530/ERC-25-0420

**Published:** 2026-01-30

**Authors:** Ziqiang Wang, Xue Song, Tingting Wang, Yangyang Xie, Qiwei Du, Weijun Zhang, Nian Liu, Rongguo Li, Jiawei He

**Affiliations:** ^1^ The First People’s Hospital of Xiaoshan District, Xiaoshan Affiliated Hospital of Wenzhou Medical University, Hangzhou, China; ^2^ Department of Respiratory and Critical Care Medicine, Hangzhou TCM Hospital Affiliated to Zhejiang Chinese Medical University, Hangzhou, China; ^3^ Department of General Practice, The Second Affiliated Hospital and Yuying Children’s Hospital of Wenzhou Medical University, Wenzhou, China; ^4^ Zhejiang Key Laboratory of Multi-Omics Precision Diagnosis and Treatment of Liver Diseases, Department of General Surgery, Sir Run-Run Shaw Hospital, Zhejiang University School of Medicine, Hangzhou, China; ^5^ Department of Breast Surgery, Hangzhou TCM Hospital Affiliated to Zhejiang Chinese Medical University, Hangzhou, China

**Keywords:** radioactive iodine, papillary thyroid carcinoma, competing risk analysis, elderly patients, nomogram

## Abstract

The aim of this study was to quantify the cancer-specific death (CSD) benefit of radioactive iodine therapy (RAIT) in older patients with N1b differentiated thyroid carcinoma, a population designated by the ATA-2025 guideline as a ‘consider RAIT’ zone despite unproven mortality benefit, and to translate this recommendation into clinically actionable subgroups using a competing-risks framework. We used data from the Surveillance, Epidemiology, and End Results (SEER) programme (2004–2015) and analysed papillary thyroid carcinoma (PTC) cases aged ≥55 years with N1b, M0. After 1:1 propensity score matching, cumulative incidence function and Fine–Gray models evaluated associations between RAIT and CSD. Stratifications included tumour size, positive lymph node burden (PLN > 5), and ETE. A nomogram was developed for 1-, 3-, and 5-year CSD prediction, and absolute risk reduction (ARR) and number needed to treat (NNT) were estimated. Among 1,142 patients, 648 were matched (n = 324/group; median follow-up = 69 months). Five-year CSD was 14.1% without RAIT versus 5.1% with RAIT (P = 0.001), yielding ARR ≈ 9% and NNT ≈ 11. RAIT was independently protective (SHR 0.33, 95% CI: 0.14–0.76). Benefit concentrated in tumours 2–4 cm, PLN > 5, and ETE-positive strata. The nomogram showed strong discrimination and calibration. In patients aged ≥55 years with N1b PTC, RAIT confers measurable mortality benefit, most evident in 2–4 cm, PLN > 5, or ETE-positive disease. Integrating ARR, NNT, and a validated nomogram, this study converts the conceptual ‘consider RAIT’ into clinically actionable ‘RAIT-favoured’ and ‘RAIT-optional’ pathways.

## Introduction

Differentiated thyroid cancer generally carries an excellent prognosis; however, patients with lateral neck nodal metastasis (N1b) who are aged ≥ 55 years face substantially higher risks of recurrence and death, which has led to wide practice variation in postoperative radioactive iodine (RAIT) indications and intensity (1). The American Thyroid Association (ATA) 2025 guideline, within the recurrence/persistence risk framework, subdivides the traditional intermediate-risk category into intermediate-low and intermediate-high – both designated as ‘consider RAIT’ – whereas high-risk or distant metastasis cases are recommended RAIT and low-risk cases are generally not recommended RAIT. Features such as N1b involvement, higher tumour diameter, a higher number of positive lymph nodes (e.g. >5), and extrathyroidal extension (ETE) are explicitly listed as scenarios in which RAIT is more likely to be favoured (2). In parallel, the European consensus clarifies the three purposes of RAIT – remnant ablation, adjuvant therapy, and treatment of the known disease – and advocates selective use in the ‘consider RAIT’ zone (3).

‘Consider’ does not equate to proven benefit on mortality endpoints. Real-world analyses have suggested that age, nodal burden, and invasive features may modify the likelihood of benefit from RAIT, but most studies have prioritized overall survival (OS) or cancer-specific survival (CSS) rather than cancer-specific death (CSD) as the core endpoint; moreover, among older patients, other-cause death (OCD) has seldom been handled within a competing-risks framework, risking over- or underestimation of RAIT’s true oncologic effect (4). For example, analyses from the Surveillance, Epidemiology, and End Results (SEER) programme indicate that among older N1b patients with a higher nodal burden, RAIT benefit is more likely to be observed, yet stratification thresholds and statistical methods have been heterogeneous, limiting bedside implementability (5). Meanwhile, safety signals – including second primary malignancies in younger patients and short- to mid-term haematologic suppression and salivary/lacrimal toxicities after RAIT – have been documented, and although the underlying evidence is heterogeneous, these findings underscore the need to identify net-benefit subgroups within the ‘consider’ population (6, 7, 8, 9).

Against this background, we focused on patients with papillary thyroid carcinoma (PTC) in SEER 2004–2015 who were aged ≥ 55 years with N1b, M0 disease: treatment‐selection bias was mitigated using propensity score matching (PSM); CSD was specified as the primary endpoint and, within a competing-risks framework cumulative incidence function (CIF) with Gray’s test and the Fine–Gray subdistribution hazard model, the association between RAIT and CSD was quantified; stratification was performed according to guideline-aligned features – tumour size, positive lymph node burden (PLN > 5), and ETE – in keeping with ATA-2025 considerations favouring RAIT; and a nomogram based on significant covariates was constructed to enable individualised prediction of 1-, 3-, and 5-year CSD (4). This study addresses a methodological gap by employing a competing-risks framework with CSD as the primary endpoint, reporting absolute effect measures – absolute risk reduction (ARR) and number needed to treat (NNT) – and delivering a deployable nomogram that translates ‘consider RAIT’ into a multidisciplinary team (MDT)-ready two-tier strategy – ‘RAIT-favoured’ and ‘RAIT-optional’ (1, 2).

## Methods

### Data source and ethics

This retrospective, population-based cohort used the publicly available SEER database (SEER*Stat v8.4.5; diagnosis years 2004–2015). SEER data are de-identified; the study complied with the Declaration of Helsinki and required no additional institutional review board approval or informed consent.

### Study population: eligibility and exclusions

#### Target population

The target population included adults with PTC and N1b nodal status, aged ≥ 55 years, treated with total thyroidectomy, M0 at diagnosis, and with complete follow-up.

#### Flow

**Figure 1:**
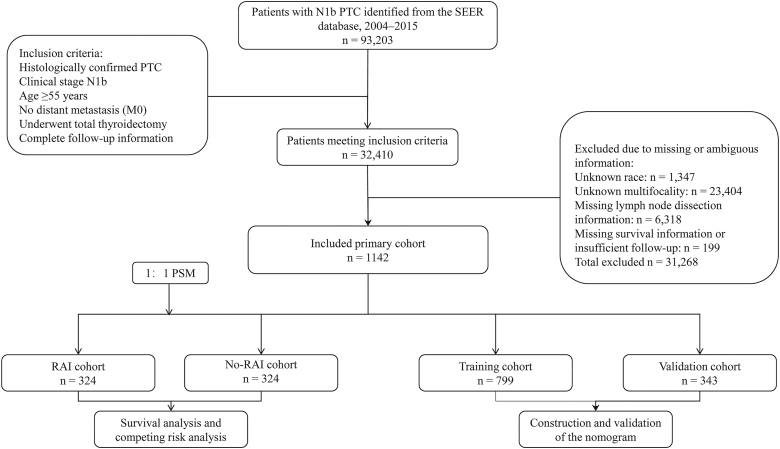
Flowchart of patient selection and study design. Abbreviations: RAIT, radioactive iodine therapy; SEER, Surveillance, Epidemiology, and End Results; PTC, papillary thyroid carcinoma; and PSM, propensity score matching.

We initially identified 93,203 PTC cases; 32,410 met core eligibility. We then excluded 31,268 for missing or inconsistent key variables: race (*n* = 1,347); multifocality (*n* = 23,404); lymph node dissection or node count (*n* = 6,318); and insufficient follow-up or survival data (*n* = 199). The final analytic cohort comprised 1,142 patients (Fig. 1).

#### Terminology

We use ‘N1b (as recorded in SEER)’ throughout. Because the SEER N category derives primarily from pathological or composite staging, we do not further distinguish cN1b from pN1b in the main text to avoid coding inconsistencies.

### Variables and definitions

#### Exposure

RAIT (yes/no). Clinicodemographic and tumour covariates. Sex; race (white vs non-white); marital status (married vs unmarried); tumour size (≤1, >1–≤2, >2–≤4, >4 cm); positive lymph node burden (PLN > 5; yes/no); ETE (yes/no); multifocality (multiple vs solitary).

#### Endpoints

OS and CSS; in the competing-risks framework: CSD and OCD.

### Propensity score matching (PSM)

were matched 1:1 using nearest-neighbour matching (calliper width 0.1 on the propensity score scale), without replacement (groups: RAIT yes/no). Matching covariates: sex, race, marital status, tumour size, PLN > 5, ETE, and multifocality. Balance was assessed using Love plots of standardised mean differences (SMDs) in conjunction with between-group tests; SMD ≤ 0.10 was taken to indicate adequate balance.

### Survival and competing-risks analyses

We generated Kaplan–Meier (KM) curves with log-rank tests for OS and CSS in the unmatched and matched cohorts. In the competing-risks framework, we estimated the CIF and compared groups with Gray’s test. In the matched cohort, we fitted Fine–Gray subdistribution-hazard models, including RAIT, sex, tumour size, PLN > 5, ETE, race, marital status, and multifocality, reporting SHR with 95% CI.

### Pre-specified subgroups

Analyses were stratified by tumour size (four levels), PLN > 5 (yes/no), and ETE (yes/no). For each stratum, we compared CIFs for CSD and OCD using Gray’s test (p1 for CSD; p2 for OCD).

### Nomogram construction and internal validation

The full cohort was randomly split 3:1 into a training set (*n* = 799) and an internal validation set (*n* = 343). Based on significant predictors from the training-set Fine–Gray model (sex, PLN > 5, treatment group, and tumour size), we constructed a nomogram for 1-, 3-, and 5-year CSD prediction. Discrimination was assessed using time-dependent AUC (time-dependent ROC) at 1, 3, and 5 years; calibration was evaluated using bootstrap calibration curves (1,000 resamples).

### Missing data and robustness

Cases with missing key variables were excluded per the flowchart (Fig. 1; complete case analysis), with potential selection bias discussed in the limitations. The primary endpoint analyses were performed in the matched cohort; the unmatched cohort is presented for context.

### Statistical considerations (time scale and censoring)

All tests were two-sided with *α* = 0.05. Analyses and visualisation were performed in R (v4.0.3) (core packages: MatchIt and optmatch for PSM; cobalt for Love plots; cmprsk for CIF and Fine–Gray; timeROC, pROC, and bootstrap for AUC and calibration).

#### Time scale

Follow-up was measured from diagnosis in months (SEER survival months).

#### Censoring rules

OS: event = death from any cause; alive/lost was right-censored at last contact.

CSS (KM): event = thyroid-cancer death; OCD treated as non-informative censoring.

Competing risks (CIF with Gray’s test and the Fine–Gray model): primary event = CSD; OCD modelled as a competing event (not censored).

Administrative censoring: primary analyses used the full SEER follow-up; results are reported at 1, 3, and 5 years, with plots to 120 months.

## Results

### Baseline characteristics

We included 1,142 patients with PTC and N1b, aged ≥ 55 years (RAIT *n* = 818; no RAIT *n* = 324) (Table 1). Overall, 79.9% were white and 63.7% were married; tumours were mainly >2–≤4 cm (30.6%) or >4 cm (19.4%); PLN > 5 was present in 51.1%, ETE in 57.7%, and multifocality in 61.7%. One-to-one PSM yielded 648 comparable patients (*n* = 324 per group). Before matching, only marital status differed (married: 65.5 vs 59.0%, *P* = 0.044). After matching, balance improved and differences were not significant for sex, marital status, tumour size, PLN > 5, ETE, and multifocality (*P* = 0.133, 0.124, 0.691, 0.208, 1.000, 0.684, respectively), with a small residual imbalance in race (white: 87.0 vs 80.9%, *P* = 0.042). Median follow-up for the full cohort was 69 months; 72 CSDs and 65 OCDs occurred. The primary endpoint model was built in the matched cohort (multivariable Fine–Gray; Table 2).

**Table 1:** Baseline characteristics of patients with N1b PTC aged ≥ 55 years treated with or without RAIT before and after PSM.

Characteristics	Before PSM			*P* value	After PSM			*P* value
	ALL	No-RAIT	RAIT		ALL	No-RAIT	RAIT	
	*n* = 1,142	*n* = 324	*n* = 818		*n* = 648	*n* = 324	*n* = 324	
Gender				0.181				0.133
Female	584 (51.1%)	155 (47.8%)	429 (52.4%)		290 (44.8%)	155 (47.8%)	135 (41.7%)	
Male	558 (48.9%)	169 (52.2%)	389 (47.6%)		358 (55.2%)	169 (52.2%)	189 (58.3%)	
Race				0.652				0.042
White	912 (79.9%)	262 (80.9%)	650 (79.5%)		544 (84.0%)	262 (80.9%)	282 (87.0%)	
Non-white	230 (20.1%)	62 (19.1%)	168 (20.5%)		104 (16.0%)	62 (19.1%)	42 (13.0%)	
Marital status				0.044				0.124
Married	727 (63.7%)	191 (59.0%)	536 (65.5%)		402 (62.0%)	191 (59.0%)	211 (65.1%)	
Unmarried	415 (36.3%)	133 (41.0%)	282 (34.5%)		246 (38.0%)	133 (41.0%)	113 (34.9%)	
Tumour size (cm)				0.947				0.691
<= 1	239 (20.9%)	69 (21.3%)	170 (20.8%)		143 (22.1%)	69 (21.3%)	74 (22.8%)	
>1–2	332 (29.1%)	90 (27.8%)	242 (29.6%)		190 (29.3%)	90 (27.8%)	100 (30.9%)	
>2–4	349 (30.6%)	101 (31.2%)	248 (30.3%)		191 (29.5%)	101 (31.2%)	90 (27.8%)	
>4	222 (19.4%)	64 (19.8%)	158 (19.3%)		124 (19.1%)	64 (19.8%)	60 (18.5%)	
LN positive				0.608				0.208
<= 5	559 (48.9%)	163 (50.3%)	396 (48.4%)		309 (47.7%)	163 (50.3%)	146 (45.1%)	
>5	583 (51.1%)	161 (49.7%)	422 (51.6%)		339 (52.3%)	161 (49.7%)	178 (54.9%)	
ETE				0.845				1.000
No	483 (42.3%)	139 (42.9%)	344 (42.1%)		278 (42.9%)	139 (42.9%)	139 (42.9%)	
Yes	659 (57.7%)	185 (57.1%)	474 (57.9%)		370 (57.1%)	185 (57.1%)	185 (57.1%)	
Focality				0.841				0.684
Multiple	705 (61.7%)	202 (62.3%)	503 (61.5%)		410 (63.3%)	202 (62.3%)	208 (64.2%)	
Single	437 (38.3%)	122 (37.7%)	315 (38.5%)		238 (36.7%)	122 (37.7%)	116 (35.8%)	

Abbreviations: RAIT, radioactive iodine therapy; PSM, propensity score matching; LN, lymph node; ETE, extrathyroidal extension; and PTC, papillary thyroid carcinoma.

**Table 2:** Multivariate Fine–Gray subdistribution hazard analysis for CSD in patients with N1b PTC aged ≥ 55 years.

Characteristics	Subdistribution proportion hazards model		
	HR	95% CI	*P* value
Gender			
Female	Reference		
Male	1.34	1.12–3.38	0.030
Race			
White	Reference		
Non-white	0.57	0.19–1.68	0.310
Marital status			
Married	Reference		
Unmarried	1.01	0.41–2.49	0.980
Tumour size (cm)			
<= 1	Reference		
>1–2	1.57	0.17–14.28	0.690
>2–4	3.24	0.36–29.6	0.300
>4	12.88	1.71–97.16	0.013
RAIT treatment			
No RAIT	Reference		
RAIT	0.33	0.14–0.76	0.009
LN positive			
<= 5	Reference		
>5	2.85	1.14–7.13	0.025
ETE			
No	Reference		
Yes	0.79	0.3–2.09	0.640
Focality			
Multiple	Reference		
Single	1.00	0.4–2.46	0.990

Abbreviations: RAIT, radioactive iodine therapy; PSM, propensity score matching; CSD, cancer-specific death; OCD, other-cause death; PTC, papillary thyroid carcinoma; CI, confidence interval; and HR, hazard ratio.

### Survival analysis

In both the unmatched cohort (OS: Supplementary Fig. S1A; CSS: Supplementary Fig. S1B (see section on Supplementary materials given at the end of the article)) and the matched cohort (OS: Supplementary Fig. S1C; CSS: Supplementary Fig. S1D), survival curves favoured RAIT, all log-rank tests: *P* < 0.001 (full figures available in the Supplementary materials).

### Competing risks

**Figure 2:**
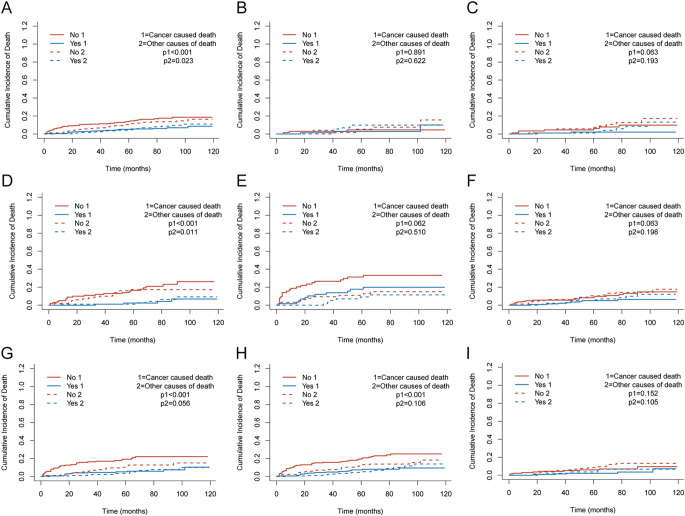
CIF curves comparing RAIT versus no RAIT for cancer-specific death (CSD) and other-cause death (OCD) in the matched cohort. (A) Overall matched cohort; (B, C, D, E) tumour-size subgroups: (B) ≤1 cm, (C) 1–≤2 cm, (D) 2–≤4 cm, (E) >4 cm; (F and G) positive lymph node (PLN) subgroups: (F) PLN ≤ 5 and (G) PLN > 5; (H and I) extrathyroidal extension (ETE) subgroups: (H) ETE positive and (I) ETE negative. Abbreviations: RAIT, radioactive iodine therapy; CIF, cumulative incidence function; CSD, cancer-specific death; OCD, other-cause death; PLN, positive lymph node; and ETE, extrathyroidal extension. A full colour version of this figure is available at https://doi.org/10.1530/ERC-25-0420.

Treating OCD as a competing event, RAIT was associated with consistently lower CSD (Fig. 2). Unmatched cohort: 1-, 3-, and 5-year CSD CIFs (no RAIT vs RAIT) were 7.5, 11.0, and 14.1 vs 0.3, 2.7, and 5.4%, respectively (Gray’s test: *P* < 0.001); the corresponding OCD CIFs were 1.2, 6.6, and 10.7 vs 0.5, 2.7, and 5.1%, respectively (*P* = 0.004). Matched cohort: 1-, 3-, and 5-year CSD CIFs were 7.5, 11.0, and 14.1% (no RAIT) vs 0.3, 3.5, and 5.1% (RAIT), respectively (*P* = 0.001); OCD CIFs were 1.2, 6.7, and 10.7 vs 0.3, 2.2, and 4.6%, respectively (*P* = 0.0226). The 5-year ARR in CSD after matching was ≈ 8.97%, corresponding to a NNT of ≈ 11–12 (see Supplementary Table S1 for detailed estimates).

### Multivariable Fine–Gray model

In the matched cohort, RAIT was independently associated with lower CSD (SHR 0.33, 95% CI: 0.14–0.76, *P* = 0.009) (Table 2). Relative to ≤1 cm, >4 cm tumours conferred higher risk (SHR 12.88, 95% CI: 1.71–97.16, *P* = 0.013), whereas 1–≤2 cm and 2–≤4 cm did not reach significance (SHR 1.57, *P* = 0.690; SHR 3.24, *P* = 0.300). PLN > 5 was an independent risk factor (SHR 2.85, 95% CI: 1.14–7.13, *P* = 0.025). Male sex was associated with higher risk (SHR 1.34, 95% CI: 1.12–3.38, *P* = 0.030). Race, marital status, ETE, and multifocality were not statistically significant (all *P* > 0.050).

### Pre-specified subgroups

Gray’s tests on stratified CIFs showed patterns directionally concordant with the matched Fine–Gray model. Tumour size: ≤1 cm (Fig. 2B) and 1–≤2 cm (Fig. 2C) were not significant or trend-level (p1 = 0.891 and 0.063 for CSD); 2–≤4 cm (Fig. 2D) showed a significant reduction in CSD (p1 < 0.001); >4 cm (Fig. 2E) was not significant (p1 = 0.062). The corresponding p2 values for OCD were 0.622, 0.193, 0.011, and 0.510, respectively. Nodal burden: PLN ≤ 5 (Fig. 2F) was not significant (p1 = 0.063), whereas PLN > 5 (Fig. 2G) showed a significant reduction (p1 < 0.001); the corresponding OCD p2 = 0.198 and 0.056. ETE: ETE positivity (Fig. 2H) showed a significant reduction (p1 < 0.001), whereas ETE negativity (Fig. 2I) was not significant (p1 = 0.152); the corresponding OCD p2 = 0.106 and 0.105 (here, p1 = Gray’s test for CSD; p2 = Gray’s test for OCD).

### Nomogram and internal validation

**Figure 3:**
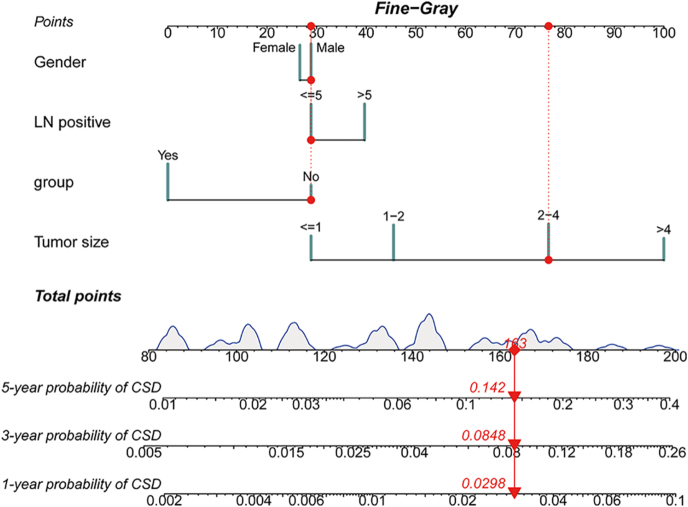
Nomogram predicting 1-, 3-, and 5-year cancer-specific death (CSD) probabilities in the matched cohort based on the Fine–Gray subdistribution-hazard model. Predictors included sex, positive lymph node burden (PLN > 5), RAIT treatment group (group: yes = received RAIT; no = did not receive RAIT), and tumour size. The total points correspond to individual 1-, 3-, and 5-year CSD probabilities. Abbreviations: RAIT, radioactive iodine therapy; CSD, cancer-specific death; and PLN, positive lymph node. A full colour version of this figure is available at https://doi.org/10.1530/ERC-25-0420.

**Figure 4:**
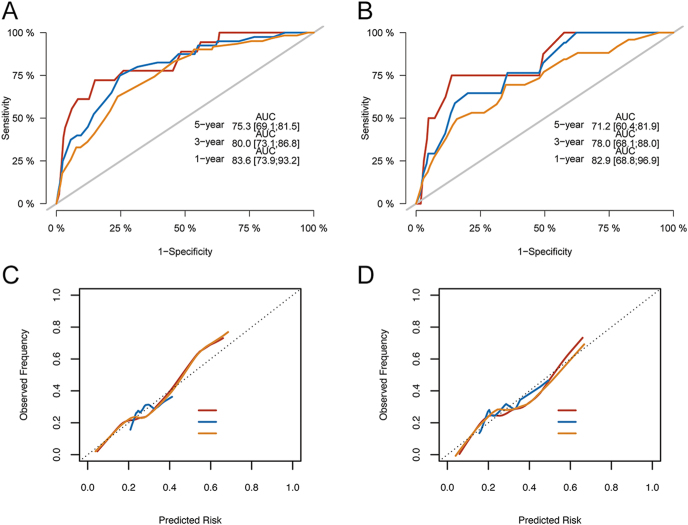
Discrimination and calibration of the Fine–Gray nomogram for predicting cancer-specific death (CSD). (A and B) Time-dependent receiver operating characteristic (ROC) curves and corresponding area under the curve (AUC) values for 1-, 3-, and 5-year CSD in the training (A) and validation (B) sets. (C and D) Calibration plots showing agreement between predicted and observed probabilities in the training (C) and validation (D) sets. Abbreviations: CSD, cancer-specific death; ROC, receiver operating characteristic; AUC, area under the curve. A full colour version of this figure is available at https://doi.org/10.1530/ERC-25-0420.

The cohort was split 3:1 into training (*n* = 799) and validation (*n* = 343) sets. A nomogram predicting 1-, 3-, and 5-year CSD was constructed from significant training-set predictors (sex, PLN > 5, treatment group, tumour size; Fig. 3). Discrimination was good: AUCs in the training set were 83.6% (1 year, 95% CI: 73.9–93.2), 80.0% (3 years, 73.1–86.8), and 75.3% (5 years, 69.1–81.5); in the validation set, AUCs were 82.9% (1 year, 68.8–96.9), 78.0% (3 years, 68.1–88.0), and 71.2% (5 years, 60.4–81.9) (Fig. 4A and B). Calibration plots showed good agreement between predicted and observed risks (Fig. 4C and D).

## Discussion

In patients aged ≥ 55 years with N1b, M0 PTC, RAIT was independently associated with lower CSD after propensity score matching (Fine–Gray SHR 0.33, 95% CI: 0.14–0.76). Within a competing-risks framework, RAIT consistently reduced the cumulative incidence of CSD across 1-, 3-, and 5-year follow-ups, corresponding to an absolute 5-year risk reduction of about 9% and a number needed to treat of ≈ 11–12. A clear concentration pattern emerged: tumours 2–≤4 cm, PLN > 5, and ETE-positive strata showed significant Gray’s tests, whereas >4 cm did not (*P* = 0.062). This distribution suggests that, in older N1b patients, nodal burden and an invasive phenotype – rather than size escalation alone – better delineate the net-benefit zone for RAIT (1, 10).

Alignment with ATA-2025 is notable. The guideline refines the intermediate-risk category into intermediate-low and intermediate-high (both ‘consider RAI’) and highlights N1b, increasing tumour size, higher numbers of positive nodes (e.g. >5), and ETE as features favouring RAIT (2, 3). Our findings are directionally concordant yet extend this framework by i) demonstrating – on a mortality endpoint using competing-risks methods – that benefit is concentrated in ≥2 cm, PLN >5, and ETE-positive subgroups, with no clear CSD gain in lower-burden strata, and ii) operationalising ‘consider’ via quantified ARR/NNT and a deployable nomogram, enabling MDT-ready stratification into ‘RAIT-favoured’ versus ‘RAIT-optional’ (4). Comparisons with prior real-world evidence support these inferences. SEER-based cohorts of N1b patients suggest that advanced age and heavy nodal burden underpin observable benefit from RAIT; some studies have proposed higher thresholds (e.g. PLN ≥ 9) to reveal such effects (5). Using a PLN > 5 versus ≤ 5 categorisation, we reproduced the same directional signal for CSD in adults aged ≥ 55 years, echoing ATA-2025’s emphasis on ‘>5’ as a burden cue favouring RAIT (2). While larger tumours are more often treated with RAIT in observational data (11), our finding of a clearer benefit at 2–≤4 cm but not at >4 cm suggests diminishing marginal explanatory power of size alone in older patients – potentially owing to higher OCD and limited events reducing statistical power, alongside unmeasured confounding (extent of surgery, RAIT activity and preparation, thyroglobulin, and imaging) not captured in SEER (12). By contrast, ETE and nodal burden are pathobiological surrogates for microscopic residuals or subclinical dissemination – the actionable targets of adjuvant RAIT (13, 14).

Beyond mortality endpoints, relapse scenarios are also relevant when interpreting RAIT in older N1b patients. Locoregional recurrence is more common in those with heavy nodal burden or ETE, features that reflect microscopic residual disease along lymphatic pathways and at the thyroid bed – the principal biological targets of adjuvant RAIT. Distant recurrence, although infrequent in differentiated PTC, increases with age and nodal burden and may further contribute to cancer-related mortality in this population. Although the SEER programme does not capture locoregional or distant relapse, the concentration of benefit we observed in patients with PLN > 5 and ETE-positive disease is biologically consistent with these relapse patterns and supports a precision approach to RAIT selection within the ATA-2025 ‘consider RAIT’ zone (15, 16).

That not all high-risk patients reached significance likely reflects the interplay between methodological and biological factors. In older adults, OCD genuinely competes with CSD in competing-risks analyses (rather than being treated as non-informative censoring); as comorbidity and treatment-related toxicity rise, the apparent oncological signal of RAIT may be partially offset (17). Although dose–toxicity data are heterogeneous and generally of low certainty, available evidence suggests that haematological suppression and salivary/lacrimal adverse effects occur within 1–12 months post-RAI and tend to increase with administered activity, supporting the use of the most tolerable (lowest effective) administered activity in those most likely to benefit (7, 8, 9). Safety signals for second primary malignancies in younger populations further underscore focussing RAIT on patients with a high probability of net benefit (6, 18).

Clinically, within the ATA-2025 ‘consider RAIT’ zone for patients aged ≥ 55 years with N1b, tumours ≥ 2 cm, PLN > 5, and ETE-positive disease constitute RAIT-favoured scenarios, in which we observed significantly lower CSDs; by contrast, tumour diameter ≤1 cm or 1–≤2 cm, PLN ≤ 5, and ETE-negative strata are RAIT-optional, warranting shared decision-making that integrates thyroglobulin, imaging, comorbidities, and patient preferences (10). With strong discrimination and calibration, the nomogram supports MDT communication of individualised 1-, 3-, and 5-year CSD risks and absolute differences with versus without RAIT (16).

Limitations are inherent to observational, registry-based analyses: residual confounding persists; SEER dataset lacks key variables (RAIT administered activity and preparation, margin status and extent of dissection, post-operative thyroglobulin, and locoregional recurrence), and differences in staging versions or recoding may introduce noise (11). The PLN > 5 versus ≤5 dichotomy precluded evaluation of higher cut-offs (e.g. ≥9); the non-significant >4 cm result may reflect sparse events together with competing mortality; and the nomogram, although internally validated, warrants external and prospective validation (19, 20, 21, 22, 23).

In summary, within the ATA-2025 ‘consider RAIT’ framework, we provide competing-risks evidence on a mortality endpoint for older patients with N1b PTC, quantify absolute effect measures – ARR and NNT, and supply a deployable nomogram that translates ‘consider’ into an actionable RAIT-favoured and RAIT-optional pathway. Future work should validate this stratification externally and prospectively, integrating RAIT activity selection and toxicity surveillance to maximise net benefit while minimising unnecessary exposure (24, 25, 26).

## Conclusion

Within the ATA-2025 ‘consider RAIT’ framework, our competing-risks analysis demonstrated that in patients aged ≥ 55 years with N1b, M0 PTC, the mortality benefit of RAIT was most evident in those with tumours 2–4 cm, PLN > 5, or ETE positivity. By integrating ARR, NNT, and a validated nomogram, this study translates the conceptual ‘consider’ into actionable ‘RAIT-favoured’ and ‘RAIT-optional’ pathways, bridging quantitative evidence and multidisciplinary decision-making. External and prospective validation is warranted to refine this precision-guided use of adjuvant RAIT.

## Supplementary materials



## Declaration of interest

The authors declare that there is no conflict of interest that could be perceived as prejudicing the impartiality of the work reported.

## Funding

This work was supported by the Hangzhou Health Science and Technology Project (Grant No. B20253339) and the Basic Public Welfare Research Program of Wenzhou Science and Technology Bureau (Grant No. Y20240475).

## Datasets analysed

The datasets analysed during the current study are available in the SEER repository (https://seer.cancer.gov/data/).
