# Supplementary material for: Defining the ATA-2025 ‘consider RAIT’ zone in older patients with N1b PTC
Source: Endocr Relat Cancer. 2026 Jan 30;33(1):e250420. doi: 10.1530/ERC-25-0420 (PMC12910568; doi:10.1530/ERC-25-0420)
Supplement: Supplementary file 1 [file supplementary_materials.pdf]

Supplementary Figure 1

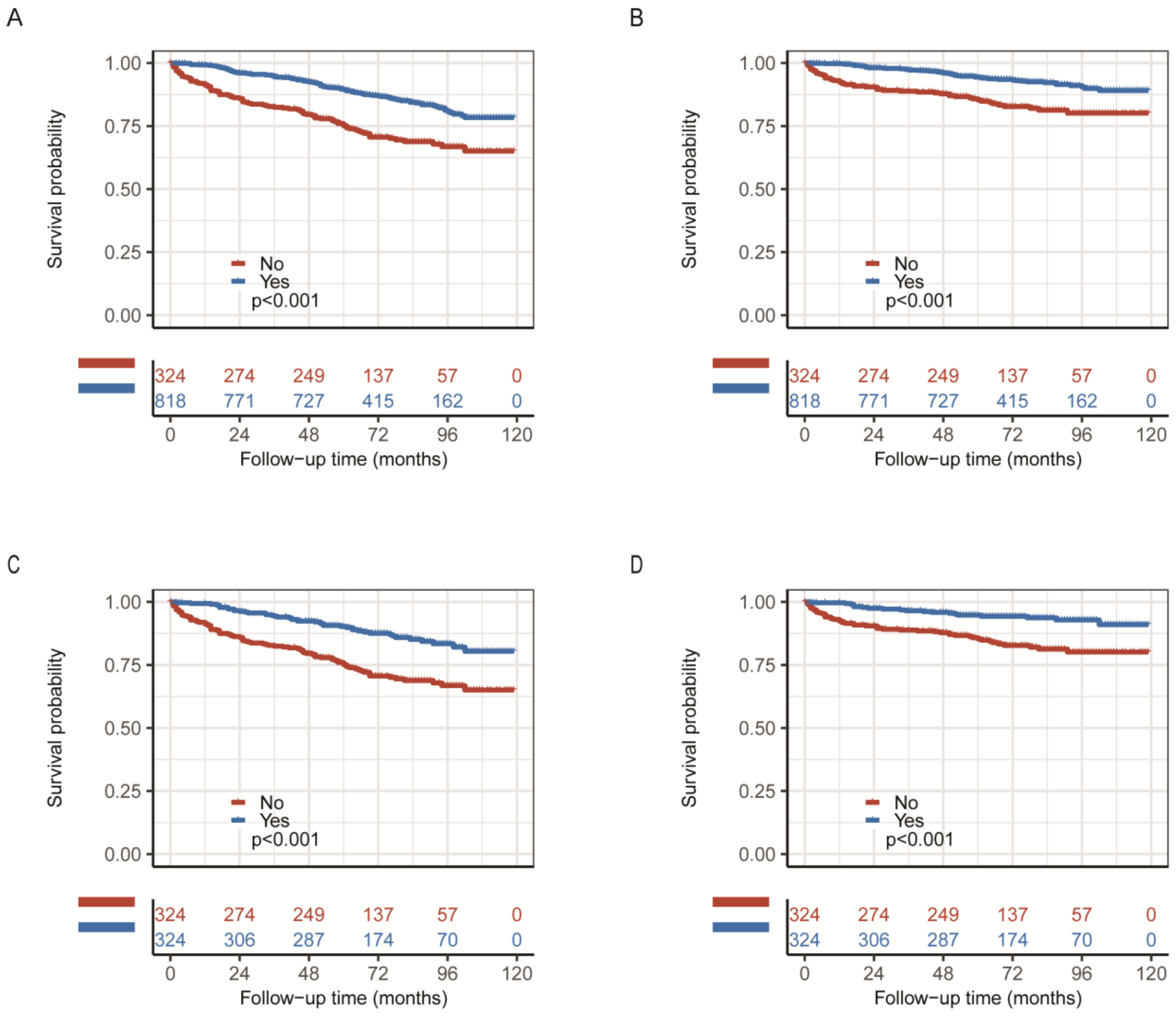

Table S1. Cumulative incidence of CSD and OCD in patients with N1b PTC aged ≥55 years from the SEER database (2004–2015), before and after PSM.

	Cancer-specific death (%)			P value	Other causes death (%)			P value
	1-year CIF	3-year CIF	5-year CIF		1-year CIF	3-year CIF	5-year CIF	
Before PSM								
No RAIT	7.5%	11.0%	14.1%	<0.001	1.2%	6.6%	10.7%	0.004
RAIT	0.3%	2.7%	5.4%		0.5%	2.7%	5.1%	
After PSM								
No RAIT	7.5%	11.0%	14.1%	0.001	1.2%	6.6%	10.7%	0.0226
RAIT	0.3%	3.5%	5.1%		0.3%	2.2%	4.6%	

Abbreviations: RAIT, radioactive iodine therapy; PSM, propensity score matching; CSD, cancer-specific death; OCD, other cause death; SEER, Surveillance, Epidemiology, and End Results; PTC, papillary thyroid carcinoma.
